# The Role of Innovation Technology in the Rehabilitation of Patients Affected by Huntington’s Disease: A Scoping Review

**DOI:** 10.3390/biomedicines12010039

**Published:** 2023-12-22

**Authors:** Maria Grazia Maggio, Luana Billeri, Davide Cardile, Angelo Quartarone, Rocco Salvatore Calabrò

**Affiliations:** IRCCS Centro Neurolesi Bonino-Pulejo, S.S. 113 Via Palermo, C. da Casazza, 98124 Messina, Italy; mariagrazia.maggio@irccsme.it (M.G.M.); luana.billeri@irccsme.it (L.B.);

**Keywords:** Huntington’s disease, neurorehabilitation, cognitive rehabilitation, virtual reality, NIBS, physical therapy

## Abstract

Huntington’s disease is an autosomal dominant neurodegenerative disease caused by the repetition of cytosine, adenine, and guanine trinucleotides on the short arm of chromosome 4p16.3 within the Huntingtin gene. In this study, we aim to examine and map the existing evidence on the use of innovations in the rehabilitation of Huntington’s disease. A scoping review was conducted on innovative rehabilitative treatments performed on patients with Huntington’s disease. A search was performed on PubMed, Embase, Web of Science, and Cochrane databases to screen references of included studies and review articles for additional citations. Of an initial 1117 articles, only 20 met the search criteria. These findings showed that available evidence is still limited and that studies generally had small sample sizes and a high risk of bias. Regarding cognitive rehabilitation, it has emerged that VR- and PC-based methods as well as NIBS techniques are feasible and may have promising effects in individuals with Huntington’s disease. On the other hand, scarce evidence was found for cognitive and motor training that might have a slight impact on overall cognitive function in individuals with Huntington’s disease. Data show that further investigation is needed to explore the effects of innovative rehabilitation tools on cognition, especially considering that cognitive and psychiatric symptoms can precede the onset of motor symptoms by many years.

## 1. Introduction

Huntington’s disease (HD), an autosomal dominant neurodegenerative disease, is caused by the repetition of cytosine, adenine, and guanine trinucleotides on the short arm of chromosome 4p16.3 within the Huntingtin gene [1,2]. This disease generally affects individuals between the age of 30 and 50, but it can also manifest before the age of 20 as juvenile HD, which is characterized by learning difficulties and behavioral disorders at school [3]. HD diagnosis is mainly clinical, based on the observation of motor and/or cognitive and behavioral disorders in individuals with a family history of the disease, and is confirmed through DNA testing [4]. Unfortunately, there is currently no cure for the disease, and treatment focuses on managing symptoms and complications (such as pneumonia and suicide attempts) and improving the quality of life [5,6]. The latter, in fact, resulted to be strongly impacted by motor, cognitive, and psychiatric disorders related to HD. Involuntary choreic movements along with cognitive and behavioral disturbances constitute pathognomonic symptoms of pathology [7,8,9,10]. Specifically, the most common motor disorders involve involuntary movements that begin in the distal extremities, including the facial muscles, and then gradually progress to the more proximal and axial muscles, increasing in amplitude [7,8]. Over time, motor symptoms progressively worsen and may include hypokinesia with bradykinesia, dystonia, rigidity, and extremity contractures. Additionally, dysarthria and dysphagia, dystonia, tics, and cerebellar signs, such as ataxia, may occur. As for behavioral and psychiatric symptoms, they often precede motor symptoms and resemble frontal lobe dysfunction, with poor attention, impulsiveness, and irritability [10,11]. Other possible symptoms include apathy, loss of intuition and creativity, psychosis, lack of awareness, and depression, often associated with suicide attempts [11,12]. Cognitive difficulties are frequently present in HD, affecting executive functions related to organization, multitasking, and planning [13], sometimes resembling subcortical dementia with memory loss [13].

Given the considerable impact the disease has on the patient’s personal, relational, and psychophysical sphere, rehabilitation approaches are an excellent option to treat these patients. The term rehabilitation refers to a multidisciplinary approach aimed at restoring or enhancing an individual’s ability to live independently and participate fully in society following an injury, illness, or disability [14]. It encompasses various traditional approaches, including physical therapy, occupational therapy, speech therapy, and neuropsychology. However, the use of new rehabilitation technologies, such as virtual reality (VR), Pc-Based training, or innovative techniques, such as non-invasive brain stimulation (NIBS), could make a significant contribution to improving functional outcomes, as seen in other conditions [15,16,17,18].

Therefore, the aim of this scoping review is to map the existing evidence on the use of innovations in the rehabilitation of HD.

## 2. Methods

We conducted this scoping review to explore existing evidence on innovative rehabilitation in HD for improving motor and cognitive outcomes in patients. This review was registered with a DOI (https://doi.org/10.17605/OSF.IO/H46KJ) on the Open Science Framework (OSF).

### 2.1. Search Strategy

This review aimed to map existing evidence on the use of innovations in Huntington’s disease rehabilitation by selecting full-length articles. A review of currently published studies was performed in the following databases: Pubmed, Embase, Cochrane Database, and Web of Science. The search terms used included ((Huntington’s disease) AND (cognitive rehabilitation); (Huntington’s disease) AND (computer); (Huntington’s disease) AND (NIBS); (Huntington’s disease) AND (physical therapy); (Huntington’s disease) AND (robotic); (Huntington’s disease) AND (virtual reality)). All articles were initially reviewed based on titles and abstracts by three investigators (M.G.M., D.C., L.B.), who independently collected data to minimize the risk of bias (e.g., publication bias, delay bias, language bias). Full-text articles deemed suitable for the study were then read by these researchers, and in case of disagreements regarding inclusion or exclusion criteria, a final decision was made by a fourth researcher (R.S.C.).

### 2.2. Inclusion Criteria

A study was included if it described or investigated patients with Huntington’s disease treated with innovative approaches of cognitive or motor rehabilitation. Only articles written in English and published in a peer-reviewed journal were included in the review.

### 2.3. Exclusion Criteria

A study was excluded if it described theoretical models, methodological approaches, algorithms, and basic technical descriptions as well as: (i) animal studies; (ii) conference proceedings or reviews; and (iii) studies involving other neurological patients.

## 3. Results

The list of articles was refined for relevance, reviewed, and summarized, with key themes identified based on inclusion/exclusion criteria.

**Figure 1:**
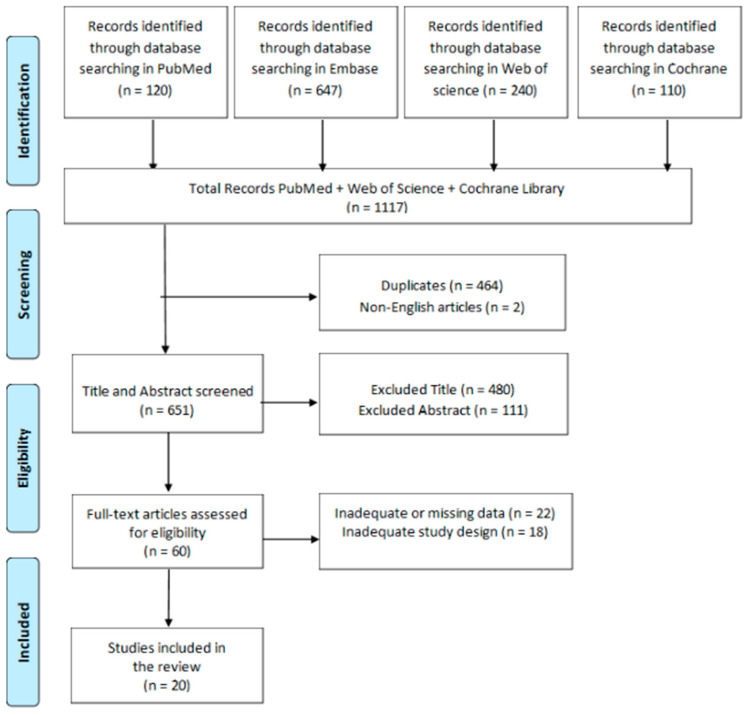
PRISMA flow chart for the current review.

Data extraction was conducted on 1117 articles. Data considered for extraction included information on authors, year and type of publication (e.g., clinical studies, pilot studies), participant characteristics, and study purposes. After a thorough review, only 20 articles met the inclusion criteria (Figure 1).

### 3.1. Innovative Rehabilitation Devices

#### 3.1.1. Virtual Reality

Virtual Reality (VR) is a multisensory and interactive simulation of ecological scenarios. These scenarios are typically three-dimensional, replicating objects and events to provide the users with the illusion of active interaction with the screen [19]. Additionally, VR provides audiovisual feedback in response to the subject’s movements. Due to its characteristics, VR has the potential to enhance motor and cognitive functions as well as improve well-being and treatment compliance [20]. VR experiences allow for patients to be at the center of the implemented programs, thanks to the high level of training customization [21]. Furthermore, two key concepts related to VR are immersion and presence. Immersion represents the objective sense of sensory absorption/immersion in the computer-generated, three-dimensional environment. This process is connected to the concept of presence, with a subjective psychological state in which the user is consciously engaged in the virtual context. From this perspective, VR could be highly beneficial in the treatment of cognitive, emotional, and motor disorders in HD patients.

The VR devices used in the reviewed studies differ significantly. Some devices promote daily life skills, as seen in RCT conducted by Begeti et al. [22]. The authors utilized a VR tool where patients swim in a pool using a joystick, with the task of reaching a submerged platform guided by external cues. Similarly, Julio et al. used a VR device, namely “EcoKitchen”, which enhances the executive functions necessary for simple daily tasks, such as making toast or a cup of coffee with milk, or simple actions, such as removing a kettle from heat. This highlights the potential role of VR in increasing task execution awareness [23]. Two other studies by the same authors emphasized that the “EcoKitchen” can detect executive deficits in patients with early and premanifest HD and is feasible [24,25]. Lastly, Cellini et al., in a single case study, evaluated the effectiveness of the Computer Assisted Rehabilitation Environment (CAREN), an immersive VR tool, in the rehabilitation of an HD patient, observing the potential role of VR devices in improving motor outcomes [26].

#### 3.1.2. PC-Based Rehabilitation

PC-based methods involve the use of software to implement intensive, targeted, and repeated interventions for specific cognitive and motor functions. These interventions typically include the execution of tasks or games, often referred to as ‘serious games’, that involve various domains [26]. The primary goal of this method is to enhance performance by utilizing positive reinforcement, which includes providing sensory and motivational feedback [27]. Additionally, these exercises offer therapists the flexibility to adjust the duration and difficulty of the tasks to match the individual’s characteristics and needs [27,28]. In our analysis of the literature, we identified 378 articles related to PC-based methods for interventions in HD patients.

We have selected eight studies that meet the inclusion criteria, including three RCTs.

Kempnich et al., in an RCT using a computerized program known as the MicroExpression Training Tool, found that emotion recognition training has proven promising in maintaining participant engagement [29]. Another RCT by Kloos et al. demonstrates the feasibility and effectiveness of a PC game called “Dance Dance Revolution” in patients with HD for enhancing motor skills [30]. In this context, Yhnell et al. observed that the Cogmed computer program, designed for cognitive and motor improvement, is well-received and feasible for individuals with HD [31]. The same tool has been used by Sadeghi et al. to enhance working memory with positive results [32]. On the other hand, Coulson et al. conducted a study on the messages exchanged by patients in online chats, noting that social support provided through online IT platforms plays a crucial role in patients with HD [33]. Moreover, some researchers have also emphasized the importance of multidisciplinary treatments, in which PC-based methods were included. Bartlett et al. showed that subjecting individuals with HD to a multidisciplinary intervention, including computerized training, leads to significant improvements in verbal learning, memory, attention, cognitive flexibility, and processing speed compared to conventional treatment [34]. Indeed, another study by the same authors showed that multidisciplinary rehabilitation, including PC-based approaches, can mitigate hypothalamic volume loss and sustain peripheral BDNF levels in preclinical HD individuals, improving cognitive functions [35]. Finally, Metzler-Baddeley et al. conducted a web-based survey, demonstrating that tablet-based touch screens were recognized as feasible and accessible solutions for rehabilitation with a PC app [36].

A description of the main features of the studies concerning VR and PC-based interventions can be found in Table 1.

### 3.2. Other Therapy (Cognitive and Motor Rehabilitation)

Most studies in the literature encourage the use of physical rehabilitation, either performed individually or as part of a multidisciplinary approach, to promote improvements in the physical and cognitive outcomes of HD patients [37,38,39,40,41]. Among various interventions, we have selected some articles that presented motor and cognitive therapies implemented with innovative technology (Table 2). In this context, Khalil et al. have focused on improving home-based rehabilitation with physical exercises using an exercise DVD [42]. The authors found that structured, short-term home exercise programs are practical, beneficial, and safe for individuals in the early and middle stages of HD [42].

Furthermore, Shih et al. employed a four-month coaching program in which participants utilized Fitbit devices and received support through a behavioral intervention aimed at promoting the physical activity, showing the effectiveness of this device [43].

Finally, another type of intervention with promising results is dance therapy. A study by Trinkel et al. on HD patients showed promising results in terms of spatial and bodily representations, helping to enhance motor function in individuals with HD [44].

**Table 2:** The main studies regarding other interventions in Huntington’s disease.

Studies	Study Design	Sample Size	Intervention	Device Type Tools Domains	Outcome Measures	Major Findings
Khalil et al. (2013) [42]	RCT	21 EG:11 CG:10	EG: Exercises at home three times a week for eight weeks using an exercise DVD. GC received their usual care.	DVD Motor functions	GAITRite system BBS SAM SF36	Structured, short-term home exercise programs are practical, beneficial, and safe for individuals in the early and middle stages of Huntington’s disease.
Shih et al. (2023) [43]	Clinical study	early-PD: 13 HD: 14	4-month coaching program, wore a Fitbit, and were guided through a behavioural intervention to facilitate PA uptake	Coaching Program with FitBit Motor functions	BBS	Incorporating wearables into a coaching intervention was achievable and offered valuable insights into physical activity behavior
Trinkler et al. (2019) [44]	Pilot study	19	Contemporary dance, a lyrical dance form, practiced for two hours per week over five months	Dance therapy Motor functions	UHDRS MDRS TMT LARS PBA QLI	Dance therapy has promising results in terms of spatial and bodily representations, helping to enhance motor function in individuals with HD

Legend: Berg Balance Scale (BBS), Control group (CG), Experimental Goup (EG), Unified Huntington’s Disease Rating Scale (UHDRS), Lille Apathy Rating Scale (LARS), Mattis Dementia Rating Scale (MDRS), Short Form 36 (SF36), Problem Behaviors Assessment (PBA), Quality of Life Index (QLI), Step Watch Step Activity Monitor (SAM), Trail Making Test (TMT).

### 3.3. Non-Invasive Brain Stimulation (NIBS)

Recent studies explored alterations in interhemispheric connectivity in HD and its temporal association with clinical manifestations. The prevailing understanding of motor symptoms in HD attributes them to a disorganized sensory–motor network and disrupted neurotransmission between the motor cortex and basal ganglia [45,46,47].

The use of neuromodulation techniques for managing HD-related symptoms has been FDA-approved, although their application remains primarily in the research contexts. However, growing interest surrounds non-invasive neuromodulation methods, including transcranial magnetic stimulation (TMS), transcranial electric stimulation (tES), and particularly transcranial direct current stimulation (tDCS), as potential therapies for neurodegenerative diseases, such as HD [48]. A recent systematic review highlights abnormal brain connectivity in various networks in HD, including sensory, motor, visual, and executive/attentional networks [49]. Non-invasive neuromodulation methods are believed to work by reorganizing brain networks, potentially rectifying the aberrant connectivity observed in HD, thereby positively influencing associated symptoms. While the initial studies suggest the effectiveness of these methods with minimal side effects, the evidence remains limited [50].

We evaluated two studies regarding tDCS [51,52], five studies assessing TMS [53,54,55,56,57], and two studies about tACS [58,59].

### 3.4. tDCS

The utility of transcranial electric stimulation (tES) in movement disorders has proven promising, but its mainstream application remains limited, primarily due to the diversity of patient populations and protocols [51,52]. Transcranial direct current stimulation (tDCS), combined with cognitive tasks, has shown the potential to enhance cognitive functioning, which is particularly relevant in HD, given its progressive cognitive impairment [51,52]. Two double-blinded crossover trials with tDCS have demonstrated improvements in cognitive and motor symptoms in HD, although further research is needed to understand the full extent of these effects [51,52].

### 3.5. TMS

Repetitive transcranial magnetic stimulation (rTMS) has been employed to alleviate choreiform movements in HD patients. Five studies have utilized TMS for HD [53,54,55,56,57]. These studies used different TMS modalities and coil types, targeting various brain regions. The studies reported symptom improvements, with some variations in the results likely due to different factors, including target accuracy, coil size, and pulse parameters. However, the duration of improvement, in some cases, was temporary.

### 3.6. tACS

Transcranial alternating current stimulation (tACS) is a versatile non-invasive brain stimulation (NIBS) technique that can target specific neural oscillatory frequencies, showing minimal and transient adverse effects [57,58]. Recent research has indicated that alpha frequency tACS, targeting bilateral mPFC (medial prefrontal cortex), has the potential to influence brain activity in HD patients. In summary, neuromodulation techniques, such as TMS, tDCS, and tACS, show promise in ameliorating the symptoms and connectivity issues associated with HD, but further research is necessary to better understand their efficacy and potential applications [59].

A summary of the main characteristics of studies regarding NIBS in HD patients can be found in Table 3.

## 4. Discussion

To the best of our knowledge, this is the first review that evaluated the state of the art of cognitive and motor rehabilitation using innovative methods in HD. Our review showed that the available evidence is limited, and the studies generally had small sample sizes and a high risk of bias. However, this was expected because of the low prevalence of HD, and this is why we decided to carry out a scoping review on this topic.

Regarding cognitive rehabilitation, it has emerged that VR- and PC-based methods as well as NIBS techniques are feasible and may have a promising effect in individuals with HD. This is in line with previous studies that highlighted the beneficial role in cognitive training of healthy elderly individuals, patients with mild cognitive impairment, and various neurological and dementia-related conditions [60,61,62,63], including Parkinson’s disease [64] and multiple sclerosis [65,66]. Indeed, cognitive training has been shown to improve cognitive function by promoting neuroplasticity through structural and functional changes in the brain [67]. Notably, studies on cognitive training have mostly included mixed groups of individuals with pre-manifest and manifest HD, also incorporating other conditions, such as PD, making it difficult to draw conclusions about the effects based on the disease stage. However, the results from various studies suggested that the disease stage has been identified as a barrier to participation or in the benefits obtained from rehabilitation [68,69].

Nevertheless, there was low-quality evidence suggesting that cognitive training might have a slight impact on overall cognitive function in individuals with HD. Therefore, further investigation is needed to explore the effects of innovative rehabilitation tools on cognition, especially considering that cognitive and psychiatric symptoms can precede the onset of motor symptoms by many years. In fact, although the diagnosis of HD is primarily based on motor symptoms, cognitive deficits are prevalent. During the pre-manifest phase of HD, which occurs before clinical diagnosis, approximately 40% of individuals experience mild cognitive impairment [70]. This percentage escalates up to 80% at the onset of the disease [71,72]. Furthermore, cognitive symptoms have a more substantial impact on the quality of life compared to motor and psychiatric symptoms. In this perspective, given the lack of available pharmaceutical treatments to improve or preserve cognitive function in individuals with HD, it is valuable to enhance treatment possibilities with innovative methods [73]. An interesting aspect is that combined interventions, which include both physical and cognitive exercises, lead to greater results compared to interventions focused on each treatment alone. In fact, interventions based solely on physical exercises could have reduced effects on cognition. This agrees with previous reviews that emphasized the diminished effects of solely physical interventions on cognition in both HD [74,75] and dementia [76]. This aspect should not be due to the stage of HD, as our review also includes studies involving pre-manifest participants, not just manifest patients with severe symptoms [24,25,26,27,28]. Therefore, cognitive training can boost cognitive domains, also influencing motor outcomes. In fact, a previous meta-analysis demonstrated in the elderly that a combined cognitive–motor treatment is more effective than treatments implemented individually [77]. Another interesting aspect is that innovative rehabilitation has better effects on cognitive and motor domains, compared to non-specific treatments, such as activities such as puzzles or independent exercises, as observed in the control groups of some studies [23,24,25,26,30].

Regarding the studies carried out on the motor component, the studies are limited but have encouraging results. Specifically, studies were conducted both at a preventive level and following the appearance of motor symptoms. In the latter case, these are mainly studies conducted with NIBS methods, with good effects on the improvement of choreic symptoms [52,53,54]. The heterogeneity of patients, including a mixed sample of individuals with manifest/pre-manifest Huntington’s disease and other neurodegenerative disorders, along with small sample sizes, variations in protocols used, a lack of follow-up, and the diversity of standardized tests used to assess functional outcomes, are the main limitations of the study. These hinder the definition of the role of innovative rehabilitative interventions in HD. However, general considerations can be made based on the analyses conducted. First, given the scarcity of available treatments, investing in innovative rehabilitation methods could be a viable alternative. This is especially relevant since these methods show encouraging results and can also enhance patient motivation towards treatment, as observed in other neurodegenerative populations. In addition, randomized trials on larger samples to explore the possible interaction and complementarity of these innovative approaches are needed.

In conclusion, this scoping review has highlighted the feasibility and potential efficacy of innovative rehabilitation tools in the rehabilitation of cognitive and behavioral dysfunction in patients with HD. Few data are still available on the motor outcomes. Considering the negative impact of cognitive–motor dysfunction on patients’ outcomes and the poor effect of pharmacological treatments, research on innovation technology to deal with these concerns is fundamental in order to improve the quality of life of both patients and their caregivers.

## Figures and Tables

**Table 1:** The main studies regarding innovative interventions for Huntington’s disease.

Studies	Study Design	Sample Size	Intervention	Device Type Tools and Domains	Outcome Measures	Major Findings
Begeti et al. (2016) [22]	RCT	13 HC 34 preHD 18 HD	Patients swim in a pool using a joystick, tasked with reaching a submerged platform by following external cues	VR MWM Assessment and Rehab Cognitive and motor domains	ACE-R NART	The deficit in allocentric memory reflects the hippocampal pathology in HD. Manifest HD patients experience more executive and motor difficulties.
Júlio et al. (2022) [23]	Clinical Study	19 HC 10 early-onset PD 20 early-PD 15 early HD	VR tasks presented on a desktop PC in a kitchen task related to preparing a cup of coffee and milk (task A); while carrying out task A, turning off a kettle (task B); or carrying out two exercises while preparing toast with butter (Task C).	EcoKitchen VR Assessment and Rehab Executive functions	BAD IAFAI BDI-II MoCA EHI IWRT UHDRS	Patients in the early stages of PD and HD without dementia maintain awareness of their performance in simulating daily tasks. Both HD and PD patients benefit from timely motor and cognitive interventions conducted with VR.
Júlio et al. (2019) [24]	Clinical Study	15 EarlyHD 15 PreHD 9 HC	VR tasks presented on a desktop PC in a kitchen task related to preparing a cup of coffee and milk (task A); while carrying out task A, turning off a kettle (task B); or carrying out two exercises while preparing toast with butter (Task C).	EcoKitchen VR Assessment and Feasibility Cognitive domains	MoCA BDI-II UHDRS EHI IWRT PVF Stroop Test SVF SDMT DST TMTA/B WCST	The EcoKitchen task is effective in detecting functionally significant deficits in patients with early and premanifest HD, and it is highly feasible.
Júlio et al. (2019) [25]	Clinical Study	15 EarlyHD 15 PreHD 19 HC	VR tasks presented on a desktop PC in a kitchen task related to preparing a cup of coffee and milk (task A); while carrying out task A, turning off a kettle (task B); or carrying out two exercises while preparing toast with butter (Task C).	EcoKitchen VR Assessment and Feasibility Executive functions	MoCA BDI-II UHDRS EHI IWRT PVF Stroop Test SVF SDMT DST TMTA/B WCST	The EcoKitchen task is sensitive to early executive deficits in HD.
Cellini et al. (2022) [26]	Case report	21-year-old woman juvenile HD	Immersive VR using six virtual scenarios, three sessions per week for six months, with each session lasting at least 45 min	CAREN VR Rehabilitation Motor outcomes	FESI TS BBS MRC TUG 6-WT 10-WT	The usefulness of neurorehabilitation using innovative VR technologies also extends to juvenile HD
Kempnich et al. (2017) [29]	RCT	22 13 preHD 9 Early HD	EG: Participants in the training group used the METT program twice a week for 4 weeks, and the examiner sent them email or text messages as reminders. CG: The control group was put on a waiting list and underwent training after completing the post-training session.	Pc-Based emotion recognition: “Mind Reading” “Emotion Trainer” Micro Expression Training Tool (METT) Emotional	METT ERT TASIT	Emotion recognition training through METT shows potential effectiveness in terms of maintaining participant engagement.
Kloos et al. (2013) [30]	RCT	18 HD	Participants engaged in the “Dance Dance Revolution” game under supervision, and they played the handheld game without supervision for 45 min, two days a week, over a span of six weeks	“Dance Dance” Revolution game PC-games Motor abilities and adherence to treatment	GAITRite TS FSST ASBCS WHOQOL-Bref	“Dance Dance Revolution” represents a feasiable, motivating, and secure physical activity intervention for individuals living with Huntington’s disease.
Yhnell et al. (2020) [31]	RCT	26 13 CG 13 EG	EG: 12-week executive function training intervention CG: Pen and paper-based exercise	CogTrainHD Pc-Based Cognitive and motor functions	CVF Stroop test TMT DST Tower of Hanoi SDMT WCST TUG CTTT	CogTrainHD is feasible and has good acceptability for implementing home-based cognitive training interventions
Sadeghi et al. (2017) [32]	Clinical trial	7 HD	Working Memory training program (Cogmed QM) in 5 weeks	CogTrainHD Pc-Based Working Memory	WMS WJTCA-III SDMT TMTA/B HDLT-R DKEFS	HD Patients recognize advantages from intensive working memory training.
Coulson et al. (2007) [33]	Clinical Study	793 HD	1313 messages sent online by patients to receive social support were analyzed.	online internet chat PC-Based Social functions	The messages aimed at requesting support were quantified	The exchange of information and emotional assistance plays a crucial role in HD.
Bartlett et al. (2020) [34]	Clinical study	31 18 EG 13 CG	EG: A nine-month multidisciplinary rehabilitation program CG: Standard care control group.	PC training + Aerobic exercises, dual-task training, sleep hygiene, nutritional counselling	Neurocom Smart Balance Master platform MRI HD-CAB SDMT TMTA/B HVLT-R OTS	Multidisciplinary rehabilitation is clinically beneficial for individuals with HD.
Bartlett et al. (2019) [35]	Clinical study	31 18 EG 11 CG	EG: A nine-month multidisciplinary rehabilitation program CG: No intervention.	PC training + Aerobic exercises, dual-task training, sleep hygiene, nutritional counselling	MRI HADS	Multidisciplinary rehabilitation can mitigate hypothalamic volume loss and sustain peripheral BDNF levels in preclinical HD individuals, although it may not influence circadian rhythm
Metzler-Baddeley et al. (2023) [36]	Clinical study	12 HD	A web-based survey was conducted to gather information on their accessibility requirements.	HD-DRUM app PC-based usability	Web-based survey	Tablet-based touch screens were identified as viable and user-friendly solutions for app delivery

Legend: Activity-Specific Balance Confidence Scale (ASBCS), Addenbrooke’s Cognitive Examination—Revised (ACE-R), Adults and Older Adults Functional Assessment Inventory (IAFAI), Awareness of Social Inference Test (TASIT), Beck Depression Inventory–II (BDI-II), Behavioural Assessment of Dysexecutive Syndrome battery (BADS), Berg Balance Scale (BBS), Cambridge Cognitive Neuroscience Research Panel (CCNRP), Categorical Verbal Fluency (CVF), Clinch Token Transfer Test (CTTT), Computer Assisted Rehabilitation Environment (CAREN), Computer-assisted Telephone Interview (CATI), Control Group (CG), Delis-Kaplan Executive Function System (DKEFS), Digit Span Test (DST), Disease Burden Score (DBS), Edinburgh Handedness Inventory (EHI), Emotion Recognition Task (ERT), Experimental Group (EG), Falls Efficacy Scale International (FESI), Four-Square Step Test (FSST), Hospital Anxiety and Depression Scale (HADS), Health Control (HC), Health-related Quality Of Life (HRQOL), Hopkins Verbal Learning Test-Revised (HVLT-R), Hungtington Disease (HD), Huntington’s Disease Cognitive Assessment Battery (HD-CAB), Irregular Word Reading Test (IWRT), Mattis Dementia Rating Scale (MDRS), Medical Research Council (MRC), Mini-Mental State Examination (MMSE), Montreal Cognitive Assessment (MoCA), National Adult Reading Test (NART), Neurological Disorders (Neuro-QoL), One Touch Stockings of Cambridge (OTS), Parkinson Disease (PD), Perceived Stress Scale (PSS), Phonemic Verbal Fluency (PVF), Problem Behaviors Assessment Scale (PBA-s), Semantic Verbal Fluency (SVF), Symbol Digit Modalities Test (SDMT), Timed Up And Go Test (TUG), Tinetti Scale (TS), Total Functional Capacity Scale (TFC), Trail Making Test A and B (TMT), Unified Huntington’s Disease Rating Scale (UHDRS), Virtual Reality (VR), Wechsler Memory Scales (WMS), Wisconsin Card Sorting Test (WCST), Woodcock Johnson Tests of Cognitive Ability–third edition (WJTCA-III), World Health Organization Quality of Life–Bref (WHOQOL-Bref), 6-min Walking Test (6-WT), 10-m Walking Test (10-WT).

**Table 3:** The main studies regarding NIBS in Huntington’s disease.

Studies	Study Design	Sample Size	Intervention	Type NIBS Domains	Follow-Up Period	Major Findings
Eddy et al. (2017) [51]	Crossover trials	20	Sham vs. 1.5 mA anodal tDCS on the left DLPC	tDCS Working Memory	Immediately post-Rtms	Anodal tDCS improves working memory, especially in patients with more severe motor symptoms.
Bocci et al. (2020) [52]	Crossover trials	4	Sham vs. 2 mA anodal tDCS cerebellar	tDCS Dystonia		Cerebellar direct current polarization improved motor scores.
Brusa et al. (2005) [53]	Crossover trials	8	1Hz rTMS on SMA	rTMS Choreic movement	Immediatelypost-rTMS	1 Hz rTMS improves choreic movements
Shukla et al. (2013) [54]	Case series	2	Session of bilateral 1 Hz rTMS on SMA	rTMS Choreic movement	8 months	No effects on choreic movements in severe HD.
Davis et al. (2016) [55]	Case report	1	“Deep” rTMS on SMA 1 Hz	rTMS Depression anxiety		Improvement of depression and anxiety scores following the real stimulation.
Bocci et al. (2016) [56]	Crossover trials	7	TMS connected to a standard eight-shaped focal coil	TMS	Immediately post-rTMS	Changes in ipsilateral silent period (iSP: onset latency, iSPOL, and duration, iSPD) and transcallosal conduction time (TCT).
Groiss et al. (2012) [57]	Crossover trials	8	High frequency (10 Hz), low frequency (1 Hz) and sham rTMS	rTMS Cognition depression		Improvement of mood after low frequency rTMS.
Davis et al. (2023) [58]	Clinical study	22	tACS to the medial prefrontal cortex (mPFC)	tACS Apathy and other non-motor symptoms		Alpha frequency tACS targeting bilateral mPFC flattened the aperiodic slope
Davis et al. (2023) [59]	Clinical study	17	tACS to the medial prefrontal cortex (mPFC)	tACS Apathy		CNV amplitude significantly increased in response to alpha-tACS, but not delta-tACS or sham

Legend: Dorsolateral Prefrontal Cortex (DLPC); Supplementary Motor Area (SMA); Contingent Negative Variation (CNV); medial Prefrontal Cortex (mPFC).

## Data Availability

The data that support the findings of this study are available on request from the corresponding author.

## Abbreviations

HD	Huntington’s Disease
RCT	Randomized Clinical Trials
VR	Virtual Reality
PC	Personal Computer
NIBS	Non-Invasive Brain Stimulation
mPFC	Medial Prefrontal Cortex
TMS	Transcranial Magnetic Stimulation
tES	Transcranial Electric Stimulation
tDCS	Transcranial Direct Current Stimulation
tACS	Transcranial Alternating Current Stimulation
